# Exposing the Barcoding Void: An Integrative Approach to Study Snail-Borne Parasites in a *One Health* Context

**DOI:** 10.3389/fvets.2020.605280

**Published:** 2020-12-10

**Authors:** Ruben Schols, Aspire Mudavanhu, Hans Carolus, Cyril Hammoud, Kudzai C. Muzarabani, Maxwell Barson, Tine Huyse

**Affiliations:** ^1^Laboratory of Aquatic Biology, Katholieke Universiteit Leuven Kulak, Kortrijk, Belgium; ^2^Department of Biology, Royal Museum for Central Africa, Tervuren, Belgium; ^3^Department of Biological Sciences, Bindura University of Science Education, Bindura, Zimbabwe; ^4^Laboratory of Molecular Cell Biology, Katholieke Universiteit Leuven—Vlaams Instituut voor Biotechnologie Center for Microbiology, Leuven, Belgium; ^5^Limnology Research Unit, Ghent University, Ghent, Belgium; ^6^Department of Biological Sciences, University of Zimbabwe, Harare, Zimbabwe; ^7^Department of Biological Sciences, University of Botswana, Gaborone, Botswana

**Keywords:** trematodiasis, xenomonitoring, *One Health*, barcoding, artificial lake, integrative taxonomy, taxonomic impediment, parasitology

## Abstract

Trematodes are snail-borne parasites of major zoonotic importance that infect millions of people and animals worldwide and frequently hybridize with closely related species. Therefore, it is desirable to study trematodiases in a *One Health* framework, where human and animal trematodes are considered equally important. It is within this framework that we set out to study the snail and trematode communities in four artificial lakes and an abattoir in Zimbabwe. Trematode infections in snails were detected through multiplex PCR protocols. Subsequently, we identified snails by sequencing a partial mitochondrial cytochrome c oxidase subunit I (COI) fragment, and trematodes (adults from the abattoir and larval stages detected in snails) using COI and nuclear rDNA markers. Of the 1,674 collected snails, 699 were molecularly analyzed, in which we identified 12 snail and 19 trematode species. Additionally, three parasite species were sampled from the abattoir. Merely four trematode species were identified to species level through COI-based barcoding. Moreover, identification of members of the superfamilies Opisthorchioidea and Plagiorchioidea required a phylogenetic inference using the highly conserved 18S rDNA marker, as no related COI reference sequences were present in public databases. These barcoding challenges demonstrate a severe barcoding void in the available databases, which can be attributed to the neglected status of trematodiases. Adding to this, many available sequences cannot be used as different studies use different markers. To fill this gap, more studies on African trematodes, using a standardized COI barcoding region, are desperately needed.

## Introduction

Trematodiases are important yet neglected tropical diseases, caused by trematode parasites with a multi-host life cycle, which typically involves a snail intermediate host (1). Schistosomiasis is the most studied trematodiasis and affects an estimated 190 million people and millions of domesticated animals in Africa (1–3). Trematodiases such as schistosomiasis, fascioliasis, and amphistomiasis are known to pose a severe burden on the livestock industry and wild (pseudo)ruminants by reducing growth and fertility, limiting milk production, and weakening or even killing young animals (4–6). This is showcased by estimates of financial losses that exceed US$ 800 million in Africa due to fascioliasis in cattle alone (7). Wild animals are not exempt from these diseases either. An example of such are the wild elephants of Sri Lanka, in which fascioliasis has been linked to significant population declines (6). The many knowledge gaps regarding the trematode life cycles, pathology, and epidemiology complicate effective control (1). In Africa for example, the epidemiology of amphistomiasis is not well-understood since the snail intermediate host is often unknown (1, 8). Knowledge gaps are even greater for trematodes of wildlife, where many species lack molecular reference sequences (9, 10). Additionally, hybridization between animal and human trematode species has been documented and some trematodes are of major zoonotic importance (11–13). Therefore, it is recommended to study trematodiases in a *One Health* framework, where human and animal trematodes are considered equally important to map their distributions, detect high-risk locations and improve disease control (14).

As mentioned above, many trematode species are unknown or await formal description. This can partly be explained by the *taxonomic impediment* or the lack of sufficient taxonomists and funding (15). The *taxonomic impediment* is ever more pressing in the global biodiversity crisis we experience today, where many species are lost before they are formally described (15). One way to meet these challenges is DNA barcoding, an approach that uses “Short, standardized gene regions” (16) to identify species, as this would speed up the process of species discovery and description (16, 17). Others argue that DNA barcoding in itself will not replace formal taxonomy but rather complement it, also known as *integrative taxonomy* (18, 19). This approach uses life-history traits, morphological characteristics, and molecular data to describe taxa at any level (15, 18, 19). Nevertheless, DNA barcoding is indispensable in epidemiological studies of parasitic diseases (20, 21) and life cycle characterization of trematodes (19, 21, 22). The formal taxonomic description of multi-host parasites requires the identification of all intermediate and definitive hosts, thus making the description of the diversity in these groups extremely complicated (22). Infection experiments that link larval and adult stages often have not been conducted (22) and morphological identification of larval trematodes is highly error-prone and often unattainable to genus or species level (21, 23, 24). In contrast, DNA sequences are identical across the different life cycle stages and can readily delineate taxa (19, 22). Therefore, the advantages of molecular approaches make DNA barcoding a key part of integrative taxonomy for trematodes. A useful application of DNA barcoding can be found in *molecular xenomonitoring*, where intermediate hosts are screened for parasite DNA (20, 25).

As we encountered the aforementioned issues throughout our study of snail and trematode communities in four artificial lakes in Zimbabwe, we decided to take this opportunity to pinpoint the current knowledge gaps that hinder our analysis and formulate recommendations for future studies, in addition to presenting our field results.

## Materials and Methods

### Ethical Permissions

Research permits were obtained through the University of Zimbabwe (UZ), Faculty of Science Research Ethics Subcommittee in Harare, Zimbabwe. The collection of adult flukes was upon agreement between the abattoir and UZ. Permits for export and import were issued by the head of the Permits Section of the Directorate of Veterinary Services, Dr. Uchirai Chindezwa, organized through Prof. Maxwell Barson.

### Snail Collection and Experiments

**Figure 1:**
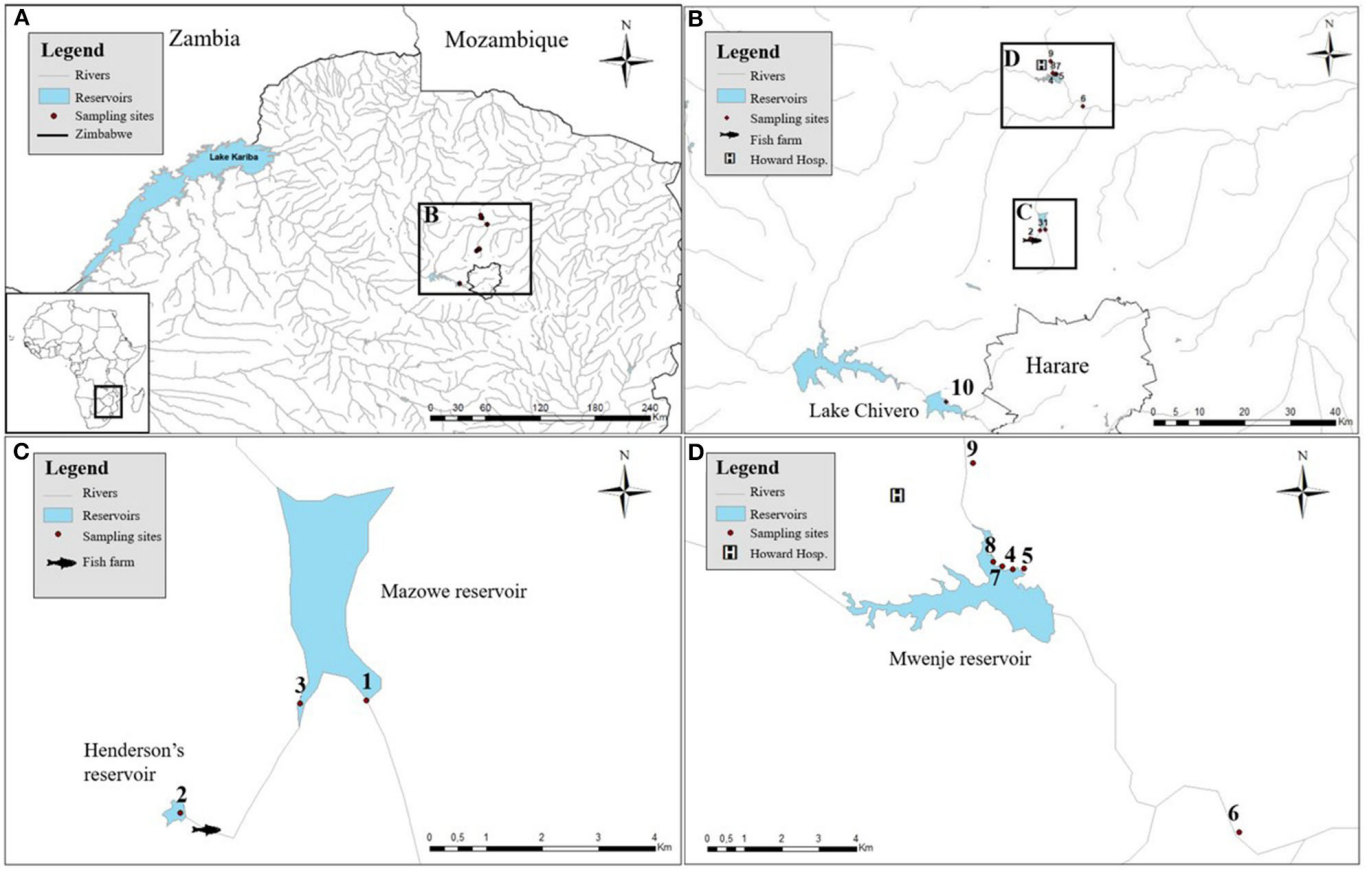
The sampled artificial lakes with the associated snail collection sites. **(A)** The locations of the sampled artificial reservoirs in Zimbabwe. **(B)** Zooming in to show their proximity to the capital (Harare). **(C)** Detailed positions of site 1 and 3 in Mazowe reservoir and site 2, upstream of a fish farm. **(D)** Detailed positions of sampling sites 4, 5, 7, and 8 in Mwenje reservoir and sites 6 and 9 down- and upstream of the reservoir, respectively.

Sampling was conducted between mid September and the beginning of October in 2018 at 10 sites spread over four artificial lakes in Zimbabwe (i.e., Henderson's, Mazowe, Mwenje, and Chivero reservoir) as shown in Figure 1. Snails were collected from aquatic vegetation and submerged substrate by hand and by scooping nets for 30 min by two persons. Collected snails were sorted per site and per morphotype, preliminarily identified according to Brown (26) and Mandahl-Barth (27) and placed individually in 12-well plates containing filtered and aged water from the site of origin. A *shedding* (i.e., release of larval trematodes) experiment was conducted to detect patent trematode infections: snails were kept in separate wells in complete darkness from 5 p.m. to 6 a.m., then exposed to bright artificial light for 6 h. Individual wells were inspected at the start and the end of the dark treatment and hourly during the light treatment. Larval parasites (cercariae) that were released from infected snails were morphologically identified based on Frandsen and Christensen (23) and fixed in 80% ethanol together with the respective snail. Non-shedding snails were pooled per species and per site for fixation and storage. High-resolution photographs were taken of all snail morphotypes per site and of each cercarial morphotype using the focus stacking system with the Zerene® stacker software (T2019-10-07-1410) as described in Brecko et al. (28).

### Adult Fluke Collection and Photographs

Adult flukes were collected from the liver and intestine of cattle at Koala park abattoir^1^ located in southern Harare. Veterinarians of the abattoir provided several flukes from randomly selected cattle. All samples were sorted according to morphotype and fixed in 70% ethanol. High-resolution photographs were taken of each morphotype using the focus stacking system of Brecko et al. (28).

### DNA Extraction

Cercarial DNA was extracted with a proteinase *K*-based lysis buffer as described in Zietara et al. (29). Adult fluke DNA was extracted from a piece of tissue isolated from the posterior end of the body with the DNeasy Blood and Tissue kit (Qiagen™) according to the manufacturer's protocol. The DNA of one snail specimen per morphotype per site was extracted with the E.Z.N.A. Mollusc DNA Kit (OMEGA Bio-tek, USA), according to Schols et al. (25). Additionally, the DNA of up to 24 additional snails per species per site (depending on snail abundance) was extracted according to the Chelex® (Biorad™) protocol described by Carolus et al. (30) to screen for pre-patent (immature) or undetected (non-shedding) patent trematode infections.

### Molecular Detection of Infected Snails

All extracted snails (*n* = 699) were screened for trematode infections through multiplex diagnostic PCR methods: planorbid and lymnaeid snails were screened according to Schols et al. (25) and Carolus et al. (30), respectively. Thiaridae were also screened according to Schols et al. (25). In short, these methods were designed to detect general trematode infections and identify *Schistosoma* spp. infections (25) and *Fasciola* spp. infections (30). Each assay includes primers that target 18S rDNA of snails as an internal control, 18S rDNA of trematodes to detect trematode infections, and the internal transcribed spacer 2 (ITS2) or a nuclear-repeat region to detect *Schistosoma* spp. and *Fasciola* spp. infections, respectively. *Schistosoma* spp. positive samples were further analyzed in a second Rapid Diagnostic (RD-)PCR according to the two-step approach described in Schols et al. (25), which identifies schistosomes of major medical and veterinary importance (i.e., *Schistosoma haematobium, S. mansoni, S. mattheei*, and *S. bovis/S. curassoni/S. guineensis*) based on variable lengths of the COI amplicon.

### PCR Protocols and DNA Sequencing

**Figure 2:**
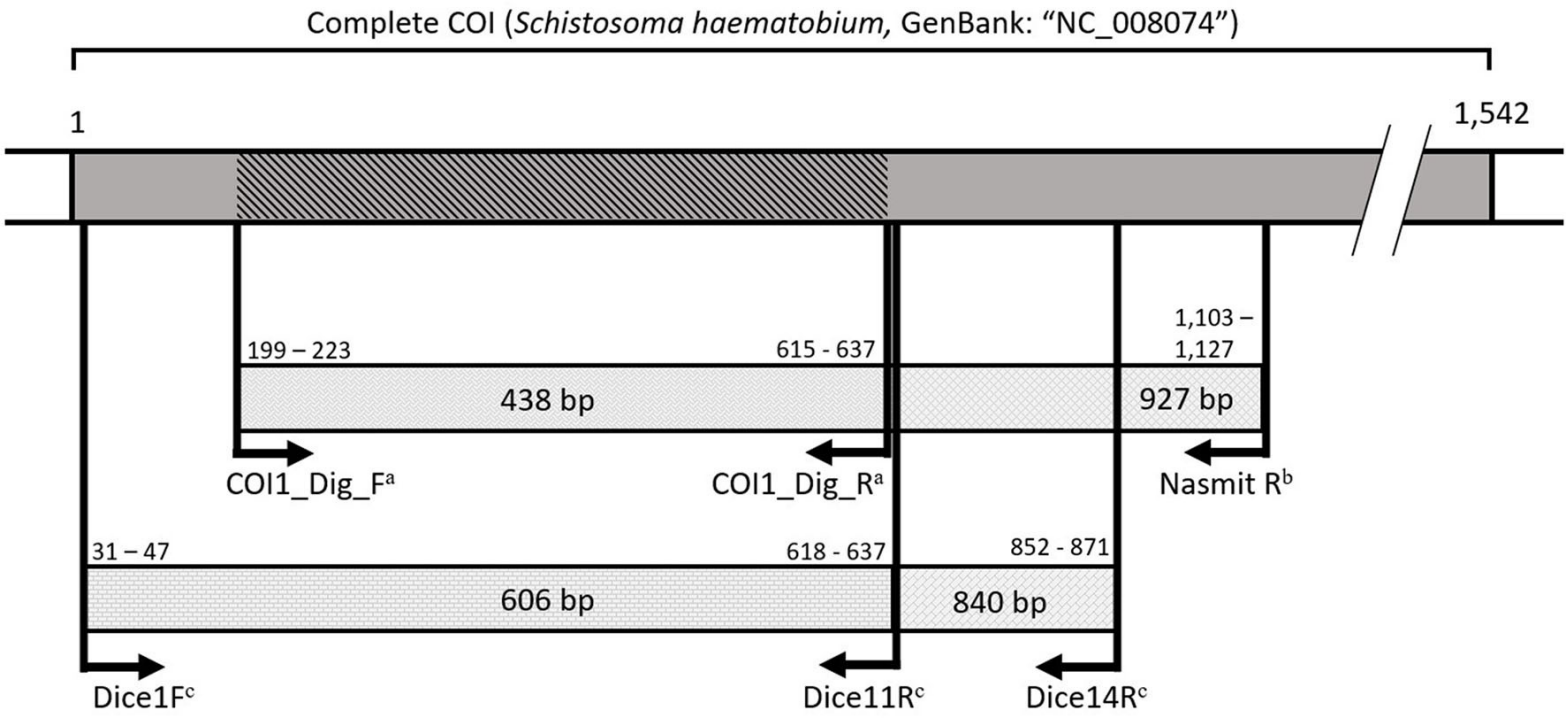
A visualization of the amplified COI fragments spanning the universal barcoding region (31) with primers from Carolus et al. (30)^a^, Hammoud et al. (in preparation)^b^, and Vansteenkiste et al. (54)^c^. All primers annealed with a maximum of one mismatch to the reference mitochondrial genome for *Schistosoma haematobium* (GenBank: “NC_008074”). Numbers indicate base pair positions in the *S. haematobium* reference COI gene. The dark gray area indicates the COI gene. The diagonally marked COI fragment indicates the shortest overlapping 438 base pairs amplified by the different primers to indicate the region used for specimen barcoding.

Based on the above-mentioned multiplex PCR protocols, at least one sample per snail species per site was selected that only showed an 18S snail internal control and an 18S trematode amplicon (thus being infected by a non-schistosome and non-fasciolid trematode). These samples were selected for sequencing following two separate PCRs using 18S rDNA and COI (respectively 1,161 and 451 bp long) to identify the trematode infection (primers listed in Supplementary Table 1). A longer COI fragment (943 bp) was targeted where possible, but amplification was often not successful. We could directly use the 18S fragment from the multiplex PCR (500 bp long), but we aimed to improve the resolution of the phylogenetic analysis with a longer alignment of the highly conserved 18S rDNA to obtain information at the (super)family level (see section Species Identification and Phylogenetic Analyses). This slowly evolving marker was not required for schistosomes and fasciolids as these groups are relatively well-covered for the ITS region in public databases. Therefore, a fragment of this region and a 451 bp fragment of the COI marker were sequenced for each schistosome or fasciolid positive snail. Initial attempts were made with general trematode rDNA primers, but if unsuccessful, we used *Fasciola* or *Schistosoma* genus-specific primers as listed in Supplementary Table 1. The snail host of each sequenced infection was barcoded using COI (536 bp). Figure 2 elaborates on the used COI primers, while all primers used for sequencing in this study are listed in Supplementary Table 1. All PCR protocols were conducted according to Carolus et al. (30) with adjusted annealing temperatures as listed in Supplementary Table 1. Amplicons of sufficient quality underwent the ExoSAP (Fermentas™) PCR purification protocol and were sequenced using forward and reverse primers at Macrogen™ (Sanger sequencing using BigDye® chemistry).

### Species Identification and Phylogenetic Analyses

All sequences were processed (i.e., quality checked, cleaned, and assembled) with Geneious® software version 10.0.9. Consensus sequences were first analyzed through a search on the GenBank^2^ and BOLD^3^ databases using the Basic Local Alignment Search Tool (BLAST) and BOLD Identification System. This outcome was used to select relevant reference sequences for phylogenetic tree construction after screening the respective submission for anomalies and selecting studies that provide morphological descriptions. The resulting selection was then aligned to our sequences with the Muscle alignment algorithm in AliView (version 1.26) (32), trimmed to the shortest sequence, and visually inspected for anomalies. Substitution model selections were performed for each alignment prior to phylogenetic analyses in MEGA version X (33) and subsequently corrected pairwise genetic distances were estimated (results not shown). The General Time Reversible (GTR) model with a discrete Gamma distribution (G) and invariable sites (I) proved suitable for all datasets, except for the 18S rDNA dataset of trematodes and COI dataset of the family Planorbidae for which we applied the Kimura 2-parameter (K2) + G model and the GTR + G model, respectively. The Maximum Likelihood (ML) trees were calculated with the selected model through the IQ-TREE web servers^4^ with 1,000 bootstraps to assess nodal support (34). A Bayesian inference of phylogeny was constructed using MrBayes (version 3.2.6) on the CIPRES portal^5^ (version 3.3) (35), using the same model, for 10,000,000 generations while sampling the Markov chain every 1,000 steps. The first 25% of the sampled values were discarded as burn-in and the analysis stopped if the convergence diagnostic fell below 0.01. FigTree^6^ was then used to combine nodal support values in a single tree.

### Voucher Specimens

All samples were curated in the RMCA museum collection following best practices in trematode systematics described by Blasco-Costa et al. (19). Samples, including snail shells; snail, larval, and adult fluke DNA extracts, and unstained tissue vouchers are all stored in the RMCA museum collection where possible, together with associated metadata, and were given unique identifiers (see Data Availability Statement).

## Results

### Snail Diversity

In total, 1,674 snails were collected across the 10 sites (Supplementary Table 2). Of these, 699 were analyzed with the above-mentioned multiplex PCR protocols (see section PCR Protocols and DNA Sequencing) whereof 77 specimens were selected for barcoding based on morphological identification and infection status. The resulting sequences were analyzed through BLAST searches, phylogenetic analyses, and pairwise distance analyses, which resulted in 12 snail species: *Bulinus globosus, Bulinus truncatus, Radix natalensis, Pseudosuccinea columella, Biomphalaria pfeifferi, Gyraulus connollyi* (?)*, Planorbella duryi, Melanoides tuberculata, Bellamya* sp., *Physella acuta*, and two *Bulinus* spp. part of the *Bulinus tropicus/truncatus* species complex (unable to be identified to species level). High-resolution photographs of each identified species can be found in Supplementary Figure 1 and phylogenetic trees of the families Planorbidae, Physidae, Lymnaeidae, and Viviparidae can be found in Supplementary Figures 2–4. Phylogenetic estimates are not shown for the family Thiaridae, because sequences were 100% identical to *M. tuberculata* representatives on GenBank (GenBank: “KP774709” from Lake Malawi and GenBank: “KF412770” from Egypt, for example) without close matches to other species. The COI corrected pairwise distances between the snail species tentatively identified as *G. connollyi* and the reference sequence of *G. connollyi* from GenBank were relatively high: 5.5 and 4.8% for haplotype 1 and 2, respectively in comparison to “KC495776” from South Africa. The corrected pairwise distances between the other species detected in this study and their matches on GenBank were below 5%, which was considered as a threshold to discriminate between intra- and interspecific variation (36, 37).

### Trematode Diversity

**Figure 3:**
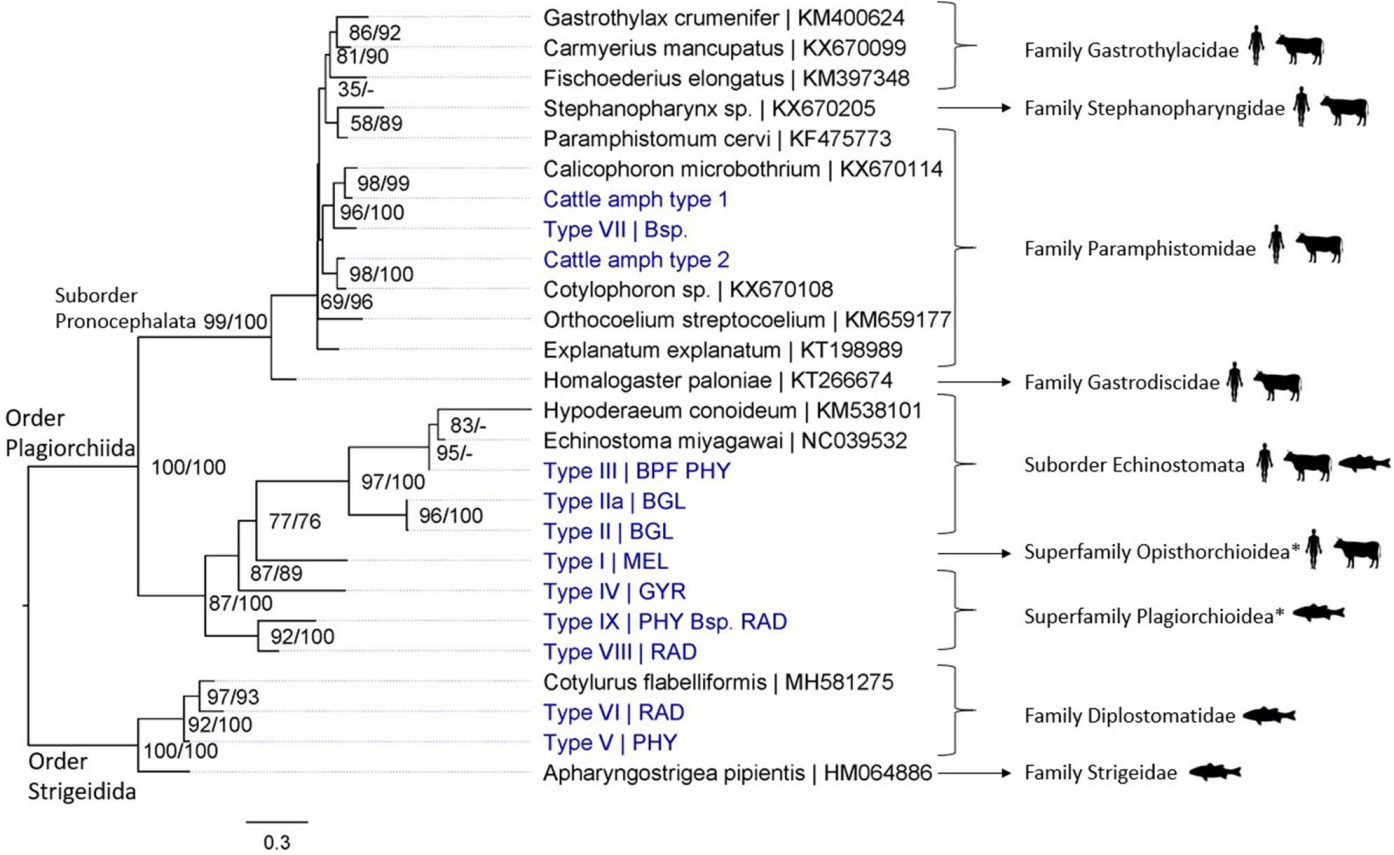
Maximum likelihood phylogenetic tree using COI mtDNA (307 bp) and using the GTR + G + I model (I = 0.18 and G = 0.45). Nodal support is indicated as bootstrap percentages (1,000 bootstraps) and posterior probabilities. Putative definitive host groups of public, veterinary, and aquaculture importance are indicated, based on host information from closely related species, with a human, ruminant, and fish icon, respectively. GenBank sequences are displayed with their accession number after the “|” separator, sequences without accession number were obtained during this study. Abbreviations of snail species that hosted the trematode species are provided after the “|” separator of each type name: MEL (*Melanoides tuberculata*), RAD (*Radix natalensis*), GYR [*Gyraulus connollyi* (?)], BPF (*Biomphalaria pfeifferi*), BGL (*Bulinus globosus*), PHY (*Physella acuta*), and Bsp. (*Bulinus* sp. 1 and 2). High-resolution pictures of released larval trematodes (cercariae) are shown in Supplementary Figure 6 of Types II, IIa, VII, and VIII. All sequences generated in this study (blue) are linked to their respective GenBank accession number and museum voucher in Supplementary Table 4. *Identification to superfamily level based on the 18S rDNA phylogenetic analysis (see Figure 4).

**Figure 4:**
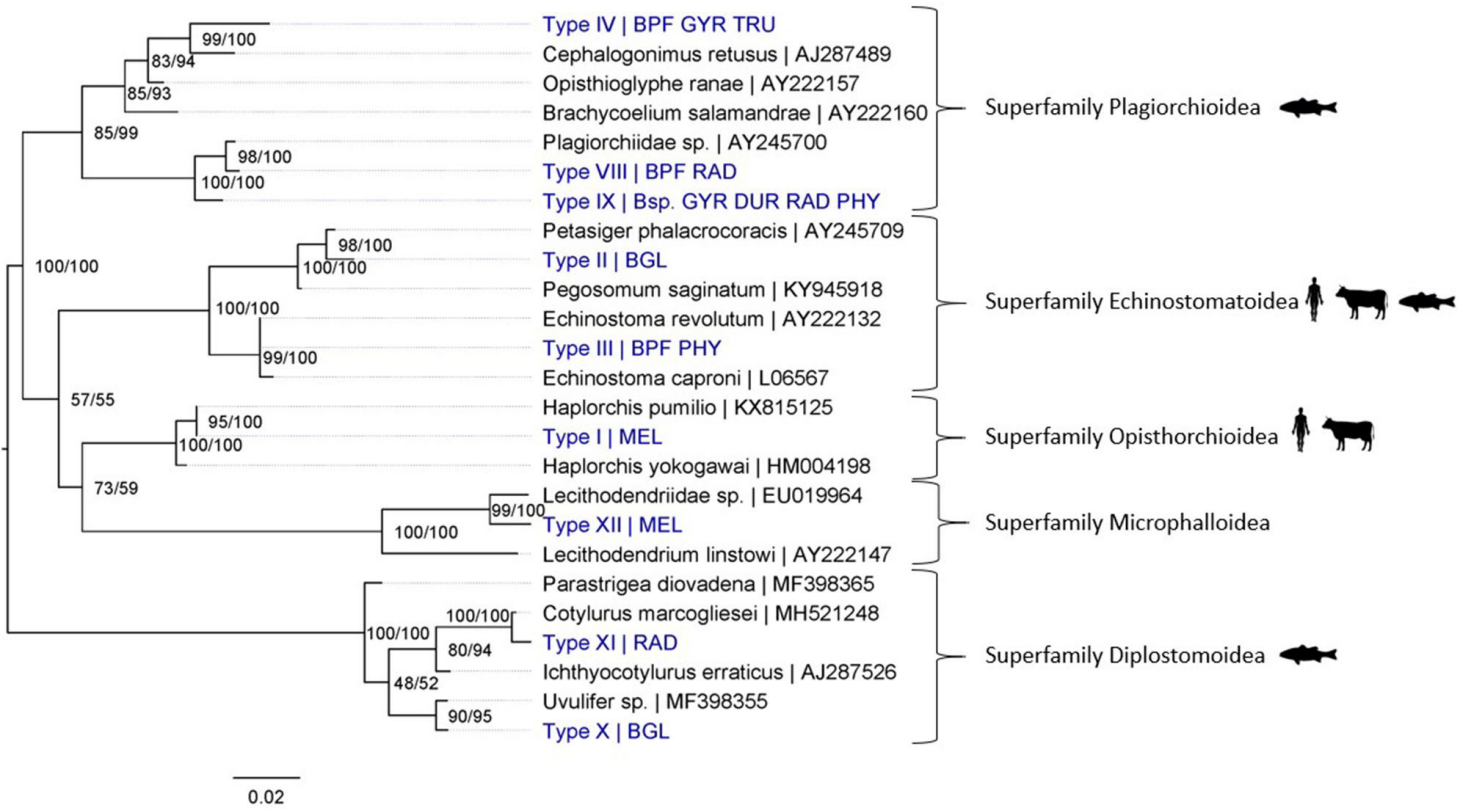
Maximum Likelihood phylogenetic tree using 18S rDNA (864 bp) and using the K2 + G model (G = 0.19). Nodal support is indicated as bootstrap percentages (1,000 bootstraps) and posterior probabilities. Putative definitive host groups of public, veterinary, and aquaculture importance are indicated, based on host information from closely related species, with a human, ruminant and, fish icon, respectively. GenBank sequences are displayed with their accession number after the “|” separator, sequences without accession number were obtained during this study. Abbreviations of snail species that hosted the trematode species are provided after the “|” separator of each type name: MEL (*Melanoides tuberculata*), RAD (*Radix natalensis*), GYR [*Gyraulus connollyi* (?)], BPF (*Biomphalaria pfeifferi*), BGL (*Bulinus globosus*), PHY (*Physella acuta*), DUR (*Planorbella duryi*), and Bsp. (*Bulinus* sp. 1 and 2). High-resolution pictures of released larval stages (cercariae) are shown in Supplementary Figure 6 of Types II and VIII. All sequences generated in this study (blue) are linked to their respective GenBank accession number and museum voucher in Supplementary Table 4.

In total, 19 different trematode species were detected in this study: 17 species from snail and cercarial DNA extracts and three from adult fluke samples (*Fasciola gigantica* was identified from both infected snails and adult flukes; Figures 3, 4). Adult flukes collected in the abattoir were grouped in three morphotypes: one was morphologically identified as *F. gigantica* based on Ashrafi et al. (38) and shown in Supplementary Figures 5C,D, the two others as amphistome species further referred to as Cattle amphistome type 1 and type 2 (Supplementary Figures 5A,B). The morphological identification of *F. gigantica* was confirmed through sequencing of COI (±800 bp) and the complete ITS region (±950 bp) (results not shown). Phylogenetic analysis indicates that “Cattle amphistome type 1” and “Cattle amphistome type 2” belong to the *Calicophoron* and *Cotylophoron* genus, respectively (Figure 3). Amphistomes are not included in the 18S rDNA phylogenetic analysis as it did not aid taxon identification.

The shedding experiments induced the release of six larval trematode morphotypes from 23 snails, which were morphologically identified as xiphidio- (Type VIII, superfamily Plagiorchioidea from *R. natalensis*), amphistome (Type VII, family Paramphistomidae from *Bulinus* sp. 2), echinostome (Type II and IIa, suborder Echinostomata from *B. globosus*), distome (failed DNA extraction) and schistosome (*S. mattheei*) cercariae based on Frandsen and Christensen (23) (Supplementary Figure 6). Supplementary Table 3 lists the trematodes that were detected per snail species per site. “Type” names missing from either of the two phylogenic analyses experienced sequencing issues. Several species required amplification of the less variable 18S rDNA region to assess their taxonomic position because closely related COI sequences were missing from the public barcoding databases (e.g., closest related sequences on GenBank exceeded 20% difference). In contrast, all species of schistosomes and fasciolids were identified to species level through mitochondrial and nuclear markers (results not shown). *Schistosoma mansoni* was detected in one out of three tested specimens of *B. pfeifferi* from site 6 (prevalence of 33%). *Schistosoma mattheei* was detected in one out of 24 tested *B. truncatus* from Henderson's reservoir, one “*Bulinus* sp. 1” out of 10 tested “*Bulinus* spp.” from Mwenje reservoir and 17 out of 137 *B. globosus* tested from Mwenje reservoir, corresponding to infection prevalences of 4.2, 10, and 12.4% respectively. Four out of 44 tested *R. natalensis* from Mwenje reservoir had *Fasciola* spp. infections (prevalence of 9.1%), two of which were barcoded as *F. gigantica* based on two ITS sequences (888 and 684 bp) and one COI sequence (683 bp). The other two were identified as *Fasciola nyanzae* as they were highly similar to the adult *F. nyanzae* from Lake Kariba, Zimbabwe collected by Schols et al. (in preparation) (99.52% identity score to GenBank: “MT909543” for 425 bp of COI; 100% identity score to GenBank: “MT909820-MT909821” for 1,055 bp of ITS). No mito-nuclear discordance, and thus no signal of hybridization, was found in the *Fasciola* and *Schistosoma* spp. sequences.

## Discussion

Molecular methods have been praised for their use in trematode species identification, matching of life cycle stages, and infection prevalence assessments (19, 39–41). Molecular species identification of trematodes relies on the availability of reference sequences in public databases. The same is true for the identification of the intermediate (snail) hosts. In the present study, we applied molecular tools to identify snail and trematode species found in four artificial lakes and a local abattoir in Zimbabwe. We combined nuclear and mitochondrial markers as this increases the chance for species identification while also uncovering possible hybridization events (11, 13).

### Host and Trematode Species Diversity

Our analyses revealed that at least 12 snail species occur in the studied artificial lakes. Molecular analysis detected DNA of at least one trematode species in 10 of the 11 screened snail species (*Bellamya* sp. was excluded, Supplementary Figures 1–4 and Supplementary Table 3). One snail species, preliminarily identified as *G. connollyi* (Supplementary Figures 1K and 2), is to our knowledge described here for the first time in Zimbabwe and North of the Limpopo river valley, which was hypothesized to form a geographical barrier for the species (42). However, only one reference sequence exists for this species (from South Africa) and the corrected pairwise distances amounted to 5%. More sequences from additional localities are needed to conclude whether this represents geographical intraspecific variation or interspecific variation. We also provide the first record of the invasive snail *P. columella* (Supplementary Figures 1I and 3) in Mwenje reservoir, but did not detect trematode infections in this snail. We found two additional exotic snails (i.e., *P. acuta* and *P. duryi*) that contained trematode DNA and therefore potentially play a role in trematode transmission. Collected native snail species include *B. pfeifferi, B. globosus, B. truncatus, M. tuberculata*, and *R. natalensis*. The endemic status of two additional *Bulinus* spp. and a *Bellamya* sp. remains uncertain as we were not able to identify the species.

Regarding trematode diversity, we identified the liver fluke *F. gigantica* and two amphistomes from the genera *Calicophoron* and *Cotylophoron* (Supplementary Figure 5), from cattle in Koala park abattoir. Our molecular analyses of larval trematode infections in snails revealed the presence of 17 trematode species (see Supplementary Table 3 for parasite species per snail species). These include *S. mattheei, S. mansoni, F. gigantica*, and *F. nyanzae*. The other trematodes, transmitted through a variety of snail species, were unidentifiable to species level and include members of the *Calicophoron* genus (*n* = 1); suborder Echinostomata (*n* = 3); superfamilies Opisthorchioidea (*n* = 1), Plagiorchioidea (*n* = 3), Microphalloidea (*n* = 1), and Diplostomoidea (*n* = 4). No hybrid trematodes were detected.

### Implications

Our molecular analyses revealed a high diversity of trematodes in snails and cattle, among which many are (potentially) important to veterinary and human health. The detected schistosomes, fasciolids, and amphistomes are all widespread in Zimbabwe and are responsible for a severe human and veterinary health burden (8, 43–47). Invasive snails, such as the detected *P. columella*, have the potential to drastically alter local trematode epidemiology (30, 48, 49). The presence of *P. columella* together with fasciolids and echinostomes warrants caution for a potential fascioliasis or echinostomiasis epidemic around Mwenje reservoir as witnessed in other areas (30, 48, 49). We also detected an unidentified trematode of the superfamily Plagiorchioidea [a group known to infect fish (1)] in what is presumably *G. connollyi*. This information combined with the known compatibility of *Gyraulus* spp. for many trematodes (50, 51), indicates that it may play a key role in disease transmission important to local fisheries and aquaculture facilities (1). Other detected trematodes are also potentially important for human and animal health, but the level of taxonomic resolution in their identification is insufficient to determine or predict affected host species.

### Bumping Up Against the Limits of Current Trematode Taxonomy

Merely four out of the 20 sequenced trematode species were identified to species level through COI-based barcoding. Nevertheless, phylogenetic inferences based on the COI marker did provide taxonomic resolution for most species to some informative level (e.g., genus or family level). However, identification of members of the superfamilies Opisthorchioidea and Plagiorchioidea required the highly conserved 18S rDNA marker, as no related species had publicly available overlapping COI reference sequences. These challenges demonstrate a severe barcoding void for trematode parasites in public databases.

The barcoding void greatly undermines the power of COI-based barcoding as an identification tool for trematodes. Based on the presence of invasive snail species and several trematode species of human and veterinary health importance, it would be advisable to monitor local transmission patterns in the studied artificial lakes. Nevertheless, a barcoding void complicates such efforts as potentially affected hosts remain unknown if the trematode taxa cannot be identified to species level. Multiple studies across the world experience similar issues [see for example (21, 22, 30) or (52)], demonstrating that the trematode barcoding void is a matter of global concern.

In this respect, molecular tools such as general or group-specific primers, a difficult hurdle for flatworms (53, 54), or xenomonitoring tools (25, 30, 55) are valuable as they can stimulate future barcoding and monitoring efforts. To ensure comparability among studies, it is paramount that newly designed primers target amplicons that overlap with existing sequences in public databases as seen in Figure 2 and stressed by Van Steenkiste et al. (54) [and see (19) for additional “Best molecular practice in trematode systematics”].

Nevertheless, integrative taxonomic approaches combining life-history traits, morphological characteristics, and molecular data will prove vital in filling the severe gap in trematode reference sequences (18, 19). These efforts are challenging as they sometimes require interdisciplinary collaborations, but they are not insurmountable [see for example (56, 57) or (58)].

To conclude, barcoding campaigns have severely neglected parasitic trematodes, but integrative taxonomic efforts and xenomonitoring studies have the potential to mitigate this knowledge gap and contribute to our understanding of trematode biology. In doing so, these efforts will aid in the description and conservation of biological diversity, and stimulate the control of trematodiases that ravage human and animal populations across Africa and the rest of the world.

## Data Availability Statement

All sequences generated in this study are deposited on NCBI GenBank: snail COI sequences (GenBank: “MT992941-MT992960 and MW205967”), trematode 18S sequences (GenBank: “MT994244-MT994253”), trematode COI sequences (GenBank: “MT994261-MT994281”) and trematode ITS sequences (GenBank: “MW046867-MW046876”). Samples, including snail shells; snail, larval, and adult fluke DNA extracts, and tissue vouchers are all stored in the RMCA museum collection, together with associated metadata, and were given unique identifiers. Isolate names, GenBank accession numbers and museum voucher numbers are all listed in Supplementary Table 4.

DNA alignments used for the analyses, sample metadata, and other additional information are available upon request from the corresponding author.

## Author Contributions

RS, HC, and TH: conceptualization. RS: formal analysis. RS, AM, and KM: investigation. MB and TH: resources. RS: data curation. RS and AM: writing—original draft. RS, AM, HC, CH, KM, MB, and TH: writing—review and editing. TH and MB: grant writing. CH, MB, and TH: supervision. All authors: contributed to the article and approved the submitted version.

## Conflict of Interest

The authors declare that the research was conducted in the absence of any commercial or financial relationships that could be construed as a potential conflict of interest.

## Footnotes

^1^www.koalaparkbutchery.com

^2^blast.ncbi.nlm.nih.gov

^3^v3.boldsystems.org

^4^iqtree.cibiv.univie.ac.at

^5^https://www.phylo.org

^6^http://tree.bio.ed.ac.uk/
