# Supplementary material for: Exposing the Barcoding Void: An Integrative Approach to Study Snail-Borne Parasites in a One Health Context
Source: Front Vet Sci. 2020 Dec 10;7:605280. doi: 10.3389/fvets.2020.605280 (PMC7758321; doi:10.3389/fvets.2020.605280)
Supplement: Supplementary file 1 [file Data_Sheet_1.docx]

Supplementary Material

# Supplementary Data

## Supplementary Figures


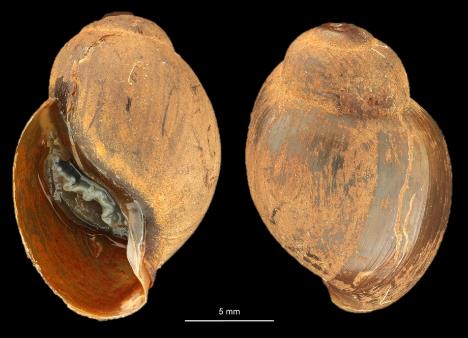

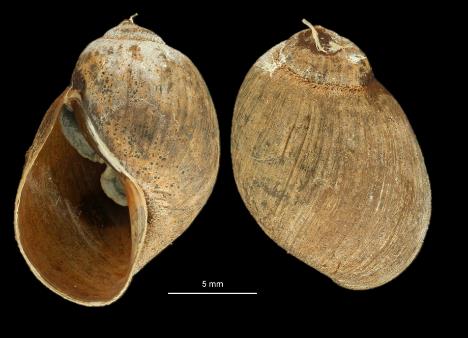

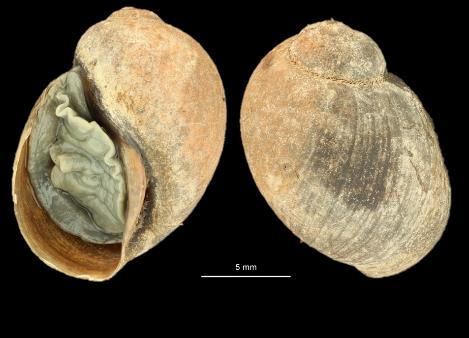

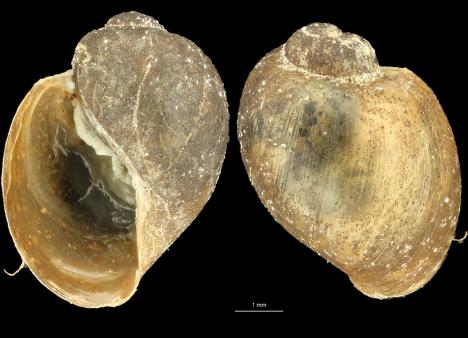

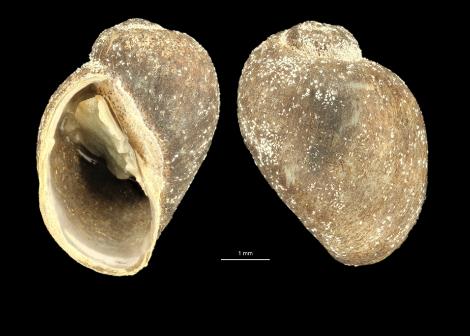

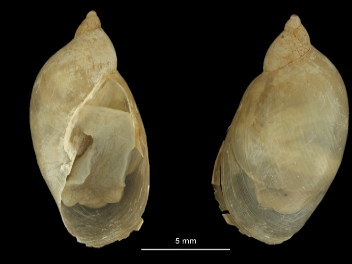

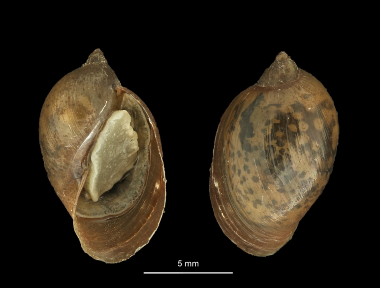

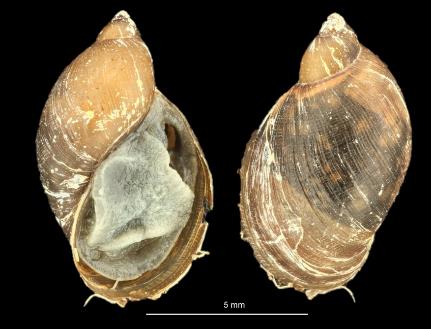

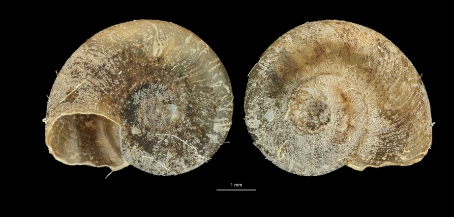

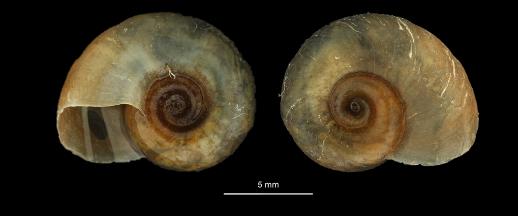

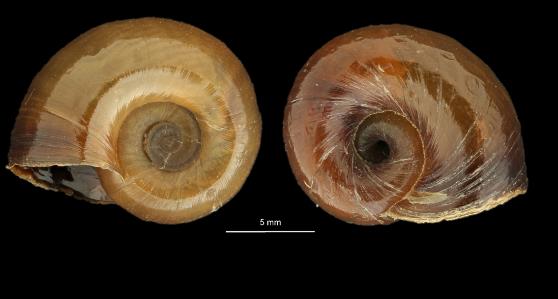

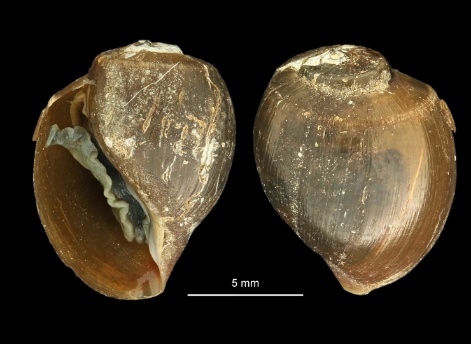


5 mm

5 mm

5 mm

5 mm

5 mm

5 mm

5 mm

5 mm

5 mm

5 mm

1 mm

1 mm

1 mm

**A1**

**B1**

**C1**

**D1**

**E1**

**F1**

**G1**

**H1**

**I1**

**J**

**K**

**L**

**M**

(continued on next page)


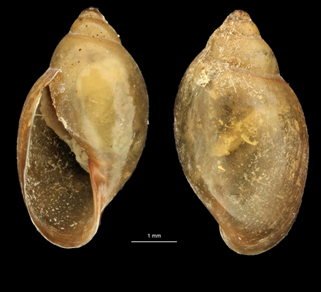


1 mm


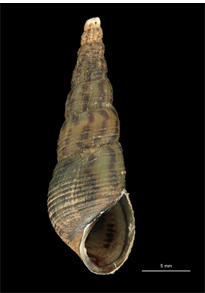

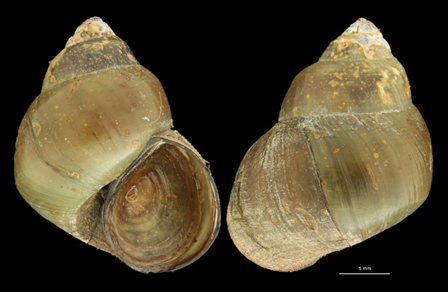


5 mm

5 mm

**O**

**N**

**P**

**Supplementary Figure 1**: All snail morphotypes collected: one representative per species is shown with the exception of *Bulinus truncatus, Bulinus globosus* and *Radix natalensis* for which two morphologically distinct but genetically similar individuals are shown. Pictures show the following species: *B. globosus* [Haplotype 1 (**A**) and haplotype 3 (**B**)], *Bulinus* sp. 1 (**C**), *Bulinus* sp. 2 (**D**), *B. truncatus* [Haplotype 1 (**E**) and haplotype 2 (**F**)], *Radix natalensis* [Haplotype 4 (**G** and **H**)], *Pseudosuccinea* *columella* (**I**), *Biomphalaria pfeifferi* (**J**), *Gyraulus connollyi* (**K**), *Planorbella duryi* (**L**), *Physella* *acuta* (**M**), *Melanoides* *tuberculata* (**N**) and *Bellamya* sp. (**O**).


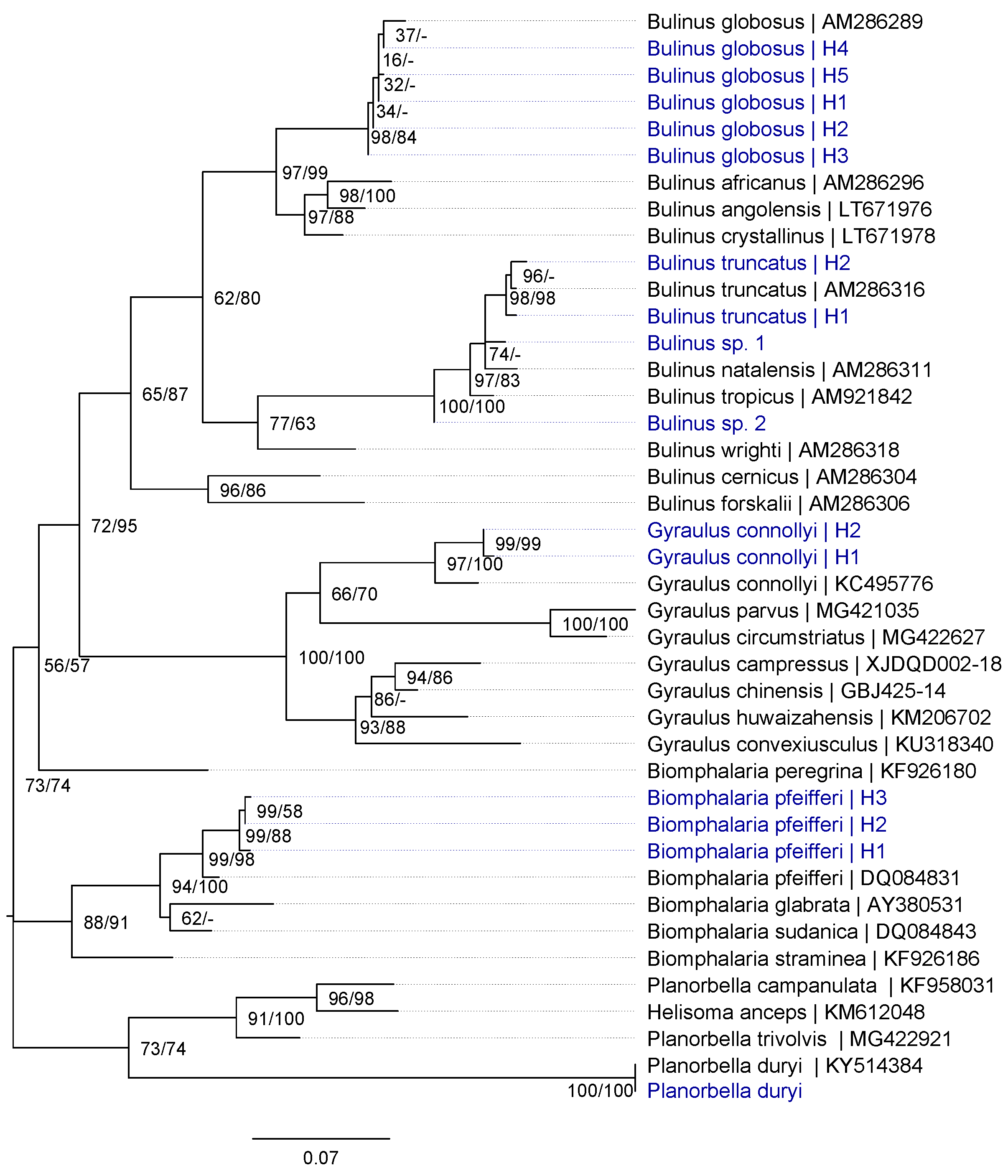


**Supplementary Figure 2**: Maximum Likelihood phylogenetic tree of the Family Planorbidae using 380 base pairs of COI mtDNA and the GTR+G model (G=0.23). Nodal support is indicated as bootstrap percentages (1,000 bootstraps) and posterior probabilities. Blue colored taxa were obtained during this study, with their haplotype (H) indicated after the ‘|’ separator. All other sequences come from GenBank with their respective accession number provided after the ‘|’ separator**.** All sequences generated in this study and indicated in the figure are linked to their respective GenBank accession number in Supplementary Table 4.


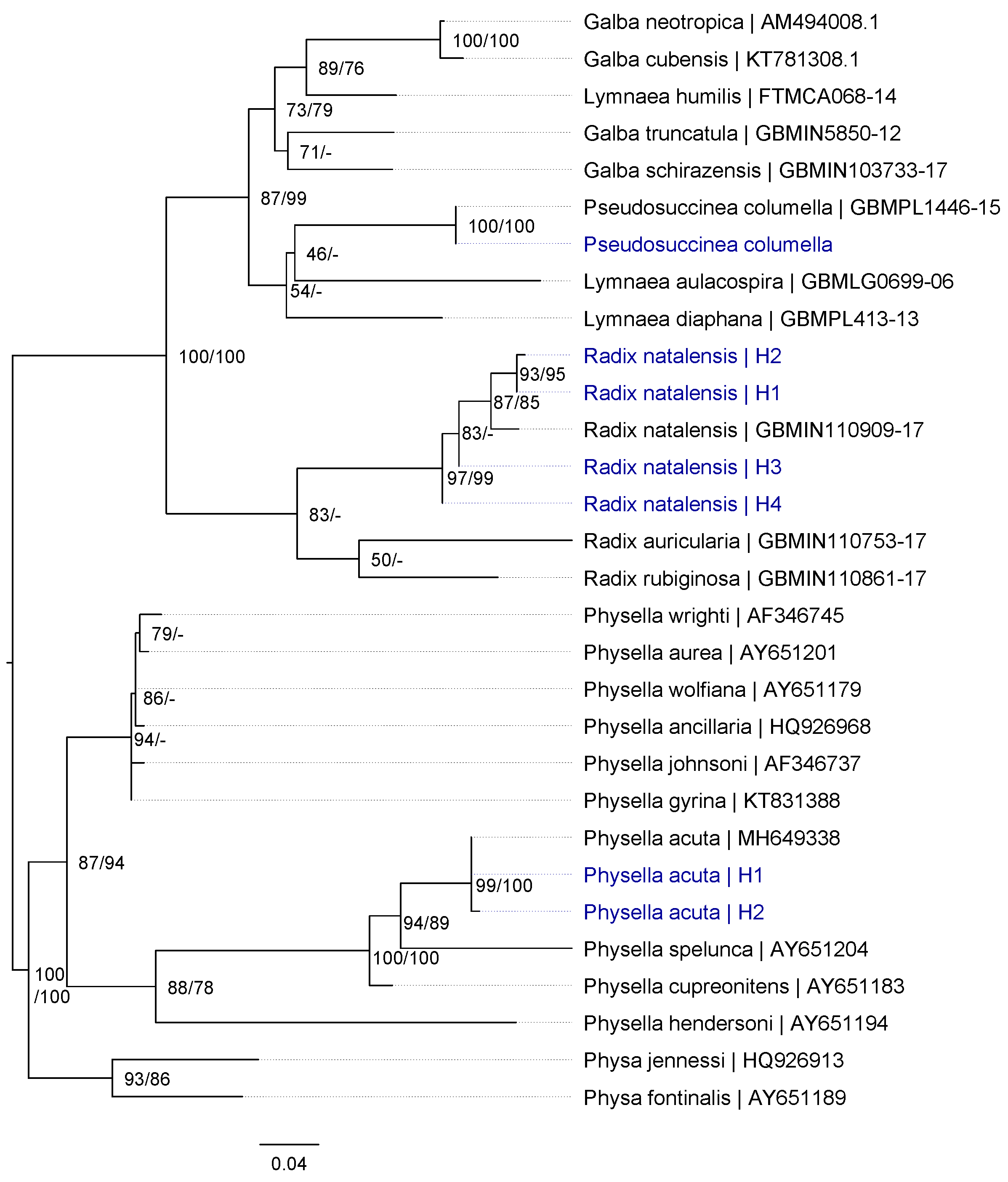


Supplementary Figure 3: Maximum Likelihood phylogenetic tree of the Family Lymnaeidae and Physidae using 463 base pairs of COI mtDNA and the GTR+G+I model (G=1.26 & I=0.53). Nodal support is indicated as bootstrap percentages (1,000 bootstraps) and posterior probabilities. Blue colored taxa were obtained during this study with their haplotype indicated after the ‘|’ separator. All other sequences come from GenBank with their respective accession number provided after the ‘|’ separator. All sequences generated in this study and indicated in the figure are linked to their respective GenBank accession number in Supplementary Table 4.


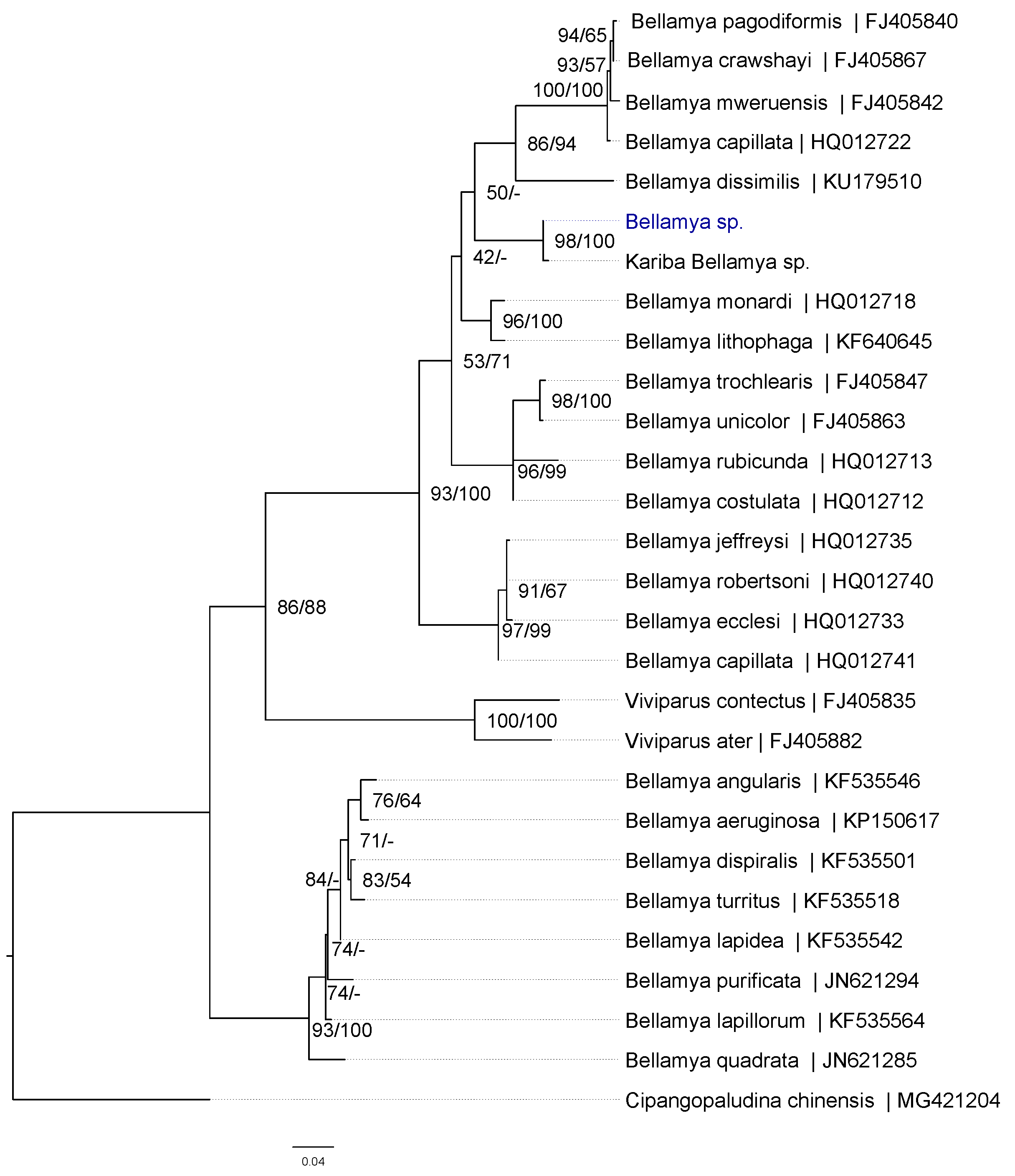


Supplementary Figure 4: Maximum Likelihood phylogenetic tree of the Viviparidae family using 393 base pairs of COI mtDNA and the GTR+G+I model (G=1.03 & I=0.47). Nodal support is indicated as bootstrap percentages (1,000 bootstraps) and posterior probabilities. Blue colored taxa were obtained during this study. All other sequences come from GenBank with their respective accession number provided after the ‘|’ separator, with the sole exception of ‘Kariba Bellamya sp.”, which was collected in an independent sampling campaign at Lake Kariba, Zimbabwe in 2017 (unpublished data). All sequences generated in this study and indicated in the figure are linked to their respective GenBank accession number in Supplementary Table 4.


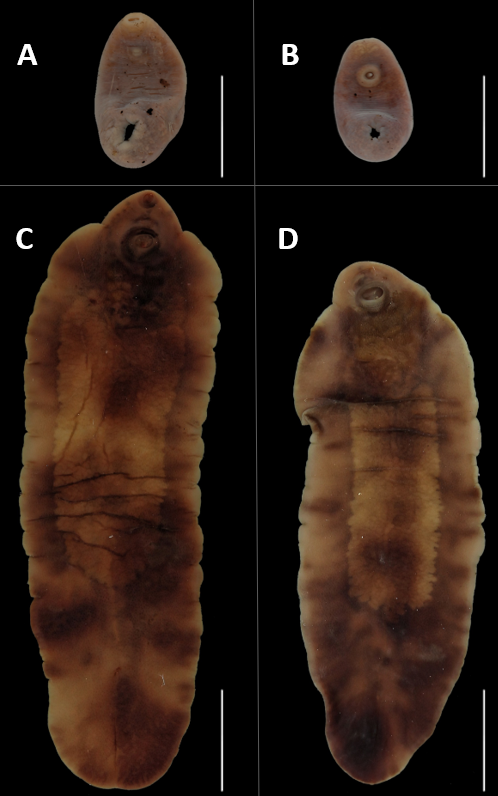


5 mm

5 mm

5 mm

5 mm

**Supplementary Figure 5**: High-definition pictures of the adult flukes collected from cattle in Koala Park abattoir, Zimbabwe. **A**) Cattle amphistome type 1, a *Calicophoron* species; **B**) Cattle amphistome type 2, a *Cotylophoron* species and **C**) & **D**) *Fasciola gigantica* specimens. **A** & **B** were collected from the intestines, **C** & **D** from the liver.


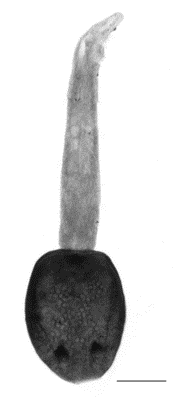

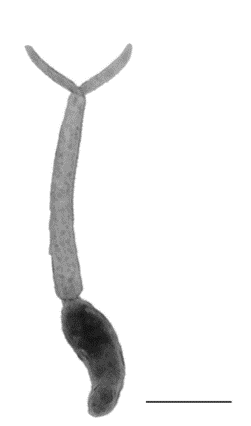

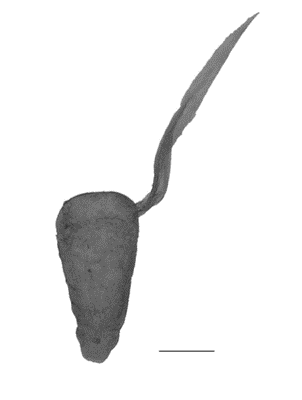

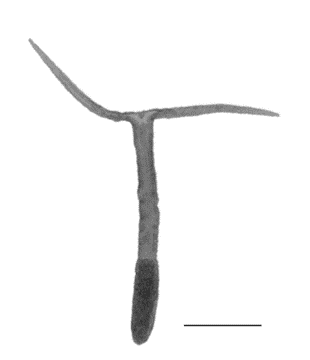

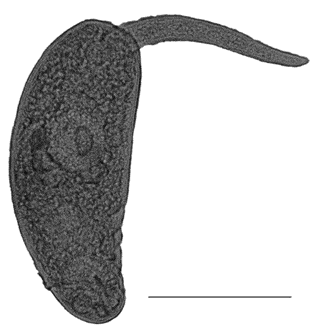

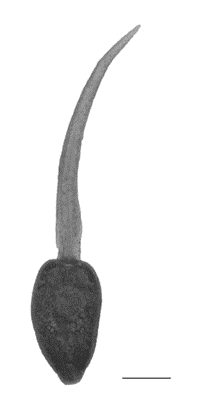


**C**

**D**

**E**

**F**

**A**

**B**

**Supplementary Figure 6:** Cercarial morphotypes identified during the shedding experiment. The following cercariae were found: **A**) Xiphidiocercaria (Type VIII, Superfamily Plagiorchioidea) from *Radix natalensis*, **B**) amphistome cercaria (Type VII, Family Paramphistomidae) from *Bulinus* sp. 2, **C**) *Schistosoma* cercaria (*S. mattheei*) from *Bulinus globosus*, **D**) Longifurcate-pharyngeate distome cercaria (failed DNA extraction) from *B. globosus*, **E**) echinostome cercaria (Type II, Suborder Echinostomata) from *B. globosus* and **F**) echinostome cercaria (Type IIa, Suborder Echinostomata) from *B. globosus*. Pictures were taken in greyscale (no staining as the specimens were used for sequencing afterwards). Scale bars represent 100 µm in each respective picture.

## Supplementary Tables

**Supplementary Table 1**: All primers used for species identification in this study organized per primer pair.

| Primer name | Used on | Marker | Annealing Temp. | Length (bp) | Primer sequence (5’-3’) | Reference |
| --- | --- | --- | --- | --- | --- | --- |
| 18S_Dig_F | trematodes | 18S | 50°C | 1,161 | CAGCTATGGTTCCTTAGATCRTA | Carolus *et al.* (2019) |
| 1270R |  |  |  |  | CCGTCAATTCCTTTAAGT | Littlewood & Olson (2001) |
| COI1_Dig_F | trematodes | COI | 53°C | 943 | CNATGATNTTNTTTTTTTTRATGCC | Hammoud *et al.* (in prep.) |
| Nasmit R |  |  |  |  | ACATAATGAAARTCAGCNAYMACRA | Hammoud *et al.* (in prep.) |
| COI1_Dig_F | trematodes | COI | 50°C | 451 | CNATGATNTTNTTTTTTTTRATGCC | Hammoud *et al.* (in prep.) |
| COI1_Dig_R |  |  |  |  | GMASWACCAAAWTTHCGATCAAA | Hammoud *et al.* (in prep.) |
| ITS2_Dig_F | trematodes | ITS2-28S | 50°C | 647 | CAAHAAGTCGTGGMTTGG | Hammoud *et al.* (in prep.) |
| ITS2_Dig_R |  |  |  |  | AACAACCCGACTCCAAGG | Hammoud *et al.* (in prep.) |
| ITS5 | trematodes | ITS1-5.8S-ITS2 | 50°C | 1,065 | GGAAGTAAAAGTCGTAACAAG | White *et al.* (1990) |
| ITS4 |  |  |  |  | TCCTCCGCTTATTGATATGC | White *et al.* (1990) |
| Fasc-ITS1 F | *Fasciola* sp. | ITS1-5.8S-ITS2 | 55°C | 716 | TCTACTCTTACACAAGCGATACAC | Grabner *et al*. (2014) |
| Fasc-ITS1 R |  |  |  |  | GGCTTTCTGCCAAGACAAG | Grabner *et al*. (2014) |
| ITS2_Schisto_F | *Schistosoma* sp. | 5.8S-ITS2 | 62°C | 369 | GGAAACCAATGTATGGGATTATTG | Schols *et al*. (2019) |
| ITS2_Schisto_R |  |  |  |  | ATTAAGCCACGACTCGAGCA | Schols *et al*. (2019) |
| COI1_snail_F | snails | COI | 50°C | 536 | TAATTWATTGTTACDGCWCATGC | Hammoud *et al.* (in prep.) |
| COI1_snail_R |  |  |  |  | CWCCTCCTGCWGGATCAAA | Hammoud *et al.* (in prep.) |

**Supplementary Table 2**: A list of the collected snail species and their numbers per collection site. The ‘sp.’ denominator in the species column is used when species identification was uncertain. Samples indicated by ‘*Bulinus* spp.’ indicate members of the *Bulinus* *tropicus/truncatus* species complex as they are morphologically indistinguishable and not all samples were genotyped.

| **Site** | **Genus** | **Species** | **Quantity** | **Site** | **Genus** | **Species** | **Quantity** |
| --- | --- | --- | --- | --- | --- | --- | --- |
| 1 | *Biomphalaria* | *pfeifferi* | 110 | 6 | *Radix* | *natalensis* | 1 |
|  | *Physella* | *acuta* | 49 |  | *Gyraulus* | *connollyi(?)* | 8 |
|  | *Radix* | *natalensis* | 63 | 7 | *Biomphalaria* | *pfeifferi* | 128 |
|  | *Bulinus* | *globosus* | 1 |  | *Physella* | *acuta* | 2 |
|  | *Bulinus* | spp*.* | 1 |  | *Radix* | *natalensis* | 10 |
| 2 | *Radix* | *natalensis* | 3 |  | *Bulinus* | *globosus* | 82 |
|  | *Bulinus* | *truncatus* | 24 |  | *Bulinus* | spp*.* | 6 |
| 3 | *Physella* | *acuta* | 41 |  | *Gyraulus* | *connollyi(?)* | 1 |
|  | *Radix* | *natalensis* | 310 |  | *Pseudosuccinea* | *columella* | 1 |
|  | *Bulinus* | *globosus* | 49 |  | *Melanoides* | *tuberculata* | 1 |
|  | *Bulinus* | spp*.* | 99 | 8 | *Biomphalaria* | *pfeifferi* | 2 |
| 4 | *Biomphalaria* | *pfeifferi* | 6 |  | *Physella* | *acuta* | 8 |
|  | *Physella* | *acuta* | 6 |  | *Radix* | *natalensis* | 28 |
|  | *Radix* | *natalensis* | 3 |  | *Bulinus* | spp*.* | 3 |
|  | *Bulinus* | *globosus* | 18 | 9 | *Biomphalaria* | *pfeifferi* | 127 |
|  | *Melanoides* | *tuberculata* | 6 |  | *Radix* | *natalensis* | 10 |
| 5 | *Biomphalaria* | *pfeifferi* | 34 |  | *Bulinus* | *globosus* | 71 |
|  | *Physella* | *acuta* | 35 |  | *Bulinus* | spp*.* | 1 |
|  | *Bulinus* | *globosus* | 136 | 10 | *Pseudosuccinea* | *columella* | 15 |
|  | *Radix* | *natalensis* | 25 |  | *Bellamya* | sp*.* | 5 |
| 6 | *Biomphalaria* | *pfeifferi* | 3 |  | *Planorbella* | *duryi* | 135 |
|  | *Physella* | *acuta* | 1 |  |  |  |  |

**Supplementary Table 3**: The detected trematode taxa per collected snail species. ‘*Bulinus* spp.’ refers to the detected members of the *Bulinus tropicus/truncatus* species complex (excluding *B. truncatus*). Collection sites of the snail intermediate host are provided in parentheses after the respective parasite name. Sites 1 and 3 were located in Mazowe reservoir; Site 2 in Henderson’s reservoir; Site 4, 5, 7 and 8 in Mwenje reservoir; Site 6 downstream and Site 9 upstream of Mwenje reservoir and Site 10 in Chivero reservoir.

| Snail host | Parasite species | | | | | |
| --- | --- | --- | --- | --- | --- | --- |
| *Radix natalensis* | Type VI (7) | Type VIII (1, 5) | Type IX (3) | Type XI (1) | *F. gigantica* (5) | *F. nyanzae* (7) |
| *Biomphalaria pfeifferi* | Type III (1) | Type IV (6) | Type VIII (5, 9) | *S. mansoni* (6) |  |  |
| *Bulinus globosus* | Type IIa (4) | Type II (4) | Type X (5) | *S. mattheei* (4, 5, 7) |  |  |
| *Physella acuta* | Type III (1) | Type V (5) | Type IX (1) |  |  |  |
| *Bulinus* spp. | Type VII (1, 3) | Type IX (3) | *S. mattheei* (7) |  |  |  |
| *Bulinus truncatus* | Type IV (7, 9) | *S. mattheei* (2) |  |  |  |  |
| *Gyraulus connollyi* | Type IV (6) | Type IX (7) |  |  |  |  |
| *Melanoides tuberculata* | Type I (4) | Type XII (4) |  |  |  |  |
| *Planorbella duryi* | Type IX (10) |  |  |  |  |  |

**Supplementary Table 4**: All sequences and vouchers generated in this study. Each unique sequence received a GenBank accession number (column ‘Acc. num.’) and is linked to the name depicted in the paper (Isolate) and the museum’s Shell/Tissue/DNA voucher numbers. The ‘xxx’ value indicates absence of the voucher number.

| Isolate | Trematode/ Snail | Acc. num. | Taxonomic identification | Marker | Shell/Tissue/DNA |
| --- | --- | --- | --- | --- | --- |
| S6-BPF-3 | trematode | MW046867 | *Schistosoma mansoni* | ITS1-5.8S-ITS2 | xxx/xxx/AB43290472 |
| S7b_BGL_1 | trematode | MW046871 | *Schistosoma mattheei* | ITS1-5.8S-ITS2 | RMCA_MOLL_804470/xxx/AB43290467 |
| Cattleamphtype1 | trematode | MW046868 | *Calicophoron* sp. | ITS1-5.8S-ITS2 | xxx/xxx/xxx |
| Cattleamphtype2 | trematode | MW046869 | *Cotylophoron* sp. | ITS1-5.8S-ITS2 | xxx/43417/xxx |
| 393 | trematode | MW046870 | *Fasciola nyanzae* | ITS1-5.8S-ITS2 | xxx/AB59864079/AB43284156 |
| 394 | trematode | MW046872 | *Fasciola nyanzae* | ITS1-5.8S-ITS2 | xxx/AB59881168/AB43284155 |
| 295 | trematode | MW046873 | *Fasciola gigantica* | ITS1-5.8S-ITS2 | xxx/AB59864036/AB43336478 |
| 296 | trematode | MW046874 | *Fasciola gigantica* | ITS1-5.8S-ITS2 | xxx/AB59864037/AB43336477 |
| F.gigantica2 | trematode | MW046875 | *Fasciola gigantica* | ITS1-5.8S-ITS2 | xxx/43412/xxx |
| F.gigantica3 | trematode | MW046876 | *Fasciola gigantica* | ITS1-5.8S-ITS2 | xxx/43413/xxx |
| S.mans_H1 | trematode | MT994261 | *Schistosoma mansoni* | COI | xxx/xxx/ AB43290472 |
| S.matt_H1 | trematode | MT994262 | *Schistosoma mattheei* | COI | xxx/xxx/AB43750572 |
| S.matt_H2 | trematode | MT994263 | *Schistosoma mattheei* | COI | xxx/xxx/AB43750595 |
| S.matt_H3 | trematode | MT994264 | *Schistosoma mattheei* | COI | xxx/xxx/AB43290467 |
| 295 | trematode | MT994265 | *Fasciola gigantica* | COI | xxx/AB59864036/AB43336478 |
| 393 | trematode | MT994266 | *Fasciola nyanzae* | COI | xxx/AB59864079/AB43284156 |
| F.gigantica1 | trematode | MT994267 | *Fasciola gigantica* | COI | xxx/43411/xxx |
| F.gigantica2 | trematode | MT994268 | *Fasciola gigantica* | COI | xxx/43412/xxx |
| F.gigantica3 | trematode | MT994269 | *Fasciola gigantica* | COI | xxx/43413/xxx |
| Cattleamphtype1 | trematode | MT994270 | *Calicophoron* sp. | COI | xxx/xxx/xxx |
| Cattleamphtype2 | trematode | MT994271 | *Cotylophoron* sp. | COI | xxx/43417/xxx |
| Type_I | trematode | MT994272 | *Opisthorchioidea* sp. | COI | xxx/xxx/AB43750590 |
| Type_II | trematode | MT994273 | *Echinostomata* sp. | COI | xxx/xxx/AB43750577 |
| Type_IIa | trematode | MT994274 | *Echinostomata* sp. | COI | xxx/xxx/AB43750567 |
| Type_III | trematode | MT994275 | *Echinostomata* sp. | COI | xxx/xxx/AB42673696 |
| Type_IV | trematode | MT994276 | *Plagiorchioidea* sp. | COI | xxx/xxx/AB43290471 |
| Type_V | trematode | MT994278 | *Diplostomidae* sp. | COI | xxx/xxx/AB43290475 |
| Type_VI | trematode | MT994279 | *Diplostomidae* sp. | COI | xxx/xxx/AB43284155 |
| Type_VII | trematode | MT994280 | *Calicophoron* sp. | COI | xxx/xxx/AB42673668 |
| Type_VIII | trematode | MT994281 | *Plagiorchioidea* sp. | COI | xxx/xxx/AB42673690 |
| Type_IX | trematode | MT994277 | *Plagiorchioidea* sp. | COI | xxx/xxx/AB42673743 |
| Type_I | trematode | MT994244 | *Opisthorchioidea* sp. | 18S | xxx/xxx/AB43750590 |
| Type_II | trematode | MT994245 | *Echinostomata* sp. | 18S | xxx/xxx/AB43750577 |
| Type_III | trematode | MT994246 | *Echinostomata* sp. | 18S | xxx/xxx/AB42673696 |
| Type_IV | trematode | MT994247 | *Plagiorchioidea* sp. | 18S | xxx/xxx/AB43290471 |
| Type_VII | trematode | MT994248 | *Calicophoron* sp. | 18S | xxx/xxx/AB42673668 |
| Type_VIII | trematode | MT994249 | *Plagiorchioidea* sp. | 18S | xxx/xxx/AB42673690 |
| Type_IX | trematode | MT994250 | *Plagiorchioidea* sp. | 18S | xxx/xxx/AB42673743 |
| Type_X | trematode | MT994251 | *Diplostomoidae* sp. | 18S | xxx/xxx/AB43750606 |
| Type_XI | trematode | MT994252 | *Diplostomoidea* sp. | 18S | xxx/xxx/AB43336542 |
| Type_XII | trematode | MT994253 | *Microphalloidea* sp. | 18S | xxx/xxx/AB43750591 |
| BGL_H1 | snail | MT992942 | *Bulinus globosus* | COI | RMCA_MOLL_804473/AB59864069/AB43750567 |
| BGL_H2 | snail | MT992943 | *Bulinus globosus* | COI | RMCA_MOLL_804471/AB59864065/AB43290456 |
| BGL_H3 | snail | MT992944 | *Bulinus globosus* | COI | RMCA_MOLL_804474/xxx/AB43341408 |
| BGL_H4 | snail | MT992945 | *Bulinus globosus* | COI | xxx/xxx/AB43750572 |
| BGL_H5 | snail | MT992946 | *Bulinus globosus* | COI | xxx/AB59864070/AB43336391 |
| BPF_H1 | snail | MT992947 | *Biomphalaria pfeifferi* | COI | xxx/xxx/AB43290472 |
| BPF_H2 | snail | MT992948 | *Biomphalaria pfeifferi* | COI | xxx/xxx/AB42673696 |
| BPF_H3 | snail | MT992941 | *Biomphalaria pfeifferi* | COI | xxx/xxx/AB43290410 |
| Bsp.1 | snail | MT992949 | *Bulinus* sp. *1* | COI | RMCA_MOLL_804457/xxx/AB43290419 |
| Bsp.2 | snail | MT992950 | *Bulinus* sp. *2* | COI | RMCA_MOLL_804454/xxx/AB42673702 |
| TRU_H1 | snail | MT992951 | *Bulinus truncatus* | COI | xxx/xxx/AB42673689 |
| TRU_H2 | snail | MT992952 | *Bulinus truncatus* | COI | xxx/xxx/AB42673685 |
| PHY_H1 | snail | MT992953 | *Physella acuta* | COI | xxx/xxx/AB43750593 |
| PHY_H2 | snail | MT992954 | *Physella acuta* | COI | xxx/xxx/AB43290475 |
| RNA_H1 | snail | MT992956 | *Radix natalensis* | COI | xxx/xxx/AB43284156 |
| RNA_H2 | snail | MT992957 | *Radix natalensis* | COI | RMCA_MOLL_804466/xxx/AB43290484 |
| RNA_H3 | snail | MT992958 | *Radix natalensis* | COI | xxx/xxx/AB42673694 |
| RNA_H4 | snail | MT992955 | *Radix natalensis* | COI | xxx/xxx/AB42673691 |
| *Bellamya* sp. | snail | MT992959 | *Bellamya* sp. | COI | xxx/AB59864038/AB42673737 |
| *Melanoides* *tuberculata* | snail | MT992960 | *Melanoides tuberculata* | COI | xxx/xxx/AB43750587 |
| *Planorbella duryi* | snail | MW205967 | *Planorbella duryi* | COI | xxx/AB59937301/AB42673721 |

## References

Carolus, H., Muzarabani, K. C., Hammoud, C., Schols, R., Volckaert, F. A. M., Barson, M., & Huyse, T. (2019). A cascade of biological invasions and parasite spillback in man-made Lake Kariba. *Science of the Total Environment*, *659*, 1283–1292. https://doi.org/10.1016/j.scitotenv.2018.12.307

Grabner, D. S., Mohamed, F. A. M. M., Nachev, M., Me, E. M. H., Sabry, A. H. A., Sures, B., Méabed, E. M. H., Sabry, A. H. A., & Sures, B. (2014). Invasion biology meets parasitology: A case study of parasite spill-back with egyptian Fasciola gigantica in the invasive snail Pseudosuccinea columella. *PLoS ONE*, *9*(2), 1–7. https://doi.org/10.1371/journal.pone.0088537

Littlewood, D. T. J., & Olson, P. D. (2001). Small subunit rDNA and the Platyhelminthes: signal, noise, conflict and compromise. *Interrelationships of the Platyhelminthes*, 262–278.

Schols, R., Carolus, H., Hammoud, C., Mulero, S., Mudavanhu, A., & Huyse, T. (2019). A rapid diagnostic multiplex PCR approach for xenomonitoring of human and animal schistosomiasis in a “One Health” context. *Transactions of the Royal Society of Tropical Medicine and Hygiene*, *113*(11), 722–729. https://doi.org/10.1093/trstmh/trz067

White, T., Bruns, T., Lee, S., Taylor, J., White, T., Lee, S., Innis, M., Gelfand, D., & Sninsky, J. (1990). Amplification and direct sequencing of fungal ribosomal RNA genes for phylogenetics. *PCR Protocols: A Guide to Methods and Applications*, *18*(1), 315–322.
